# Functional Characterization of Two Variants at the Intron 6—Exon 7 Boundary of the KCNQ2 Potassium Channel Gene Causing Distinct Epileptic Phenotypes

**DOI:** 10.3389/fphar.2022.872645

**Published:** 2022-06-13

**Authors:** Ilaria Mosca, Ilaria Rivolta, Audrey Labalme, Paolo Ambrosino, Barbara Castellotti, Cinzia Gellera, Tiziana Granata, Elena Freri, Anna Binda, Gaetan Lesca, Jacopo C. DiFrancesco, Maria Virginia Soldovieri, Maurizio Taglialatela

**Affiliations:** ^1^ Department of Medicine and Health Science “V. Tiberio”, University of Molise, Campobasso, Italy; ^2^ School of Medicine and Surgery, University of Milano-Bicocca, Monza-Center for Neuroscience (NeuroMI), Milan, Italy; ^3^ Department of Medical Genetics, Hospices Civils de Lyon, Université Claude Bernard Lyon 1, Lyon, France; ^4^ Department of Science and Technology (DST), University of Sannio, Benevento, Italy; ^5^ Unit of Medical Genetics and Neurogenetics, Fondazione IRCCS Istituto Neurologico Carlo Besta, Milan, Italy; ^6^ Department of Pediatric Neuroscience, Fondazione IRCCS Istituto Neurologico Carlo Besta, Milan, Italy; ^7^ Department of Neurology, ASST “San Gerardo” Hospital, University of Milano-Bicocca, Monza, Italy; ^8^ Department of Neuroscience, University of Naples “Federico II”, Naples, Italy

**Keywords:** alternative splicing, Kv7.2 subunits, epileptic encephalopathy, PIP_2_, calmodulin, KCNQ2

## Abstract

Pathogenic variants in KCNQ2 encoding for Kv7.2 potassium channel subunits have been found in patients affected by widely diverging epileptic phenotypes, ranging from Self-Limiting Familial Neonatal Epilepsy (SLFNE) to severe Developmental and Epileptic Encephalopathy (DEE). Thus, understanding the pathogenic molecular mechanisms of KCNQ2 variants and their correlation with clinical phenotypes has a relevant impact on the clinical management of these patients. In the present study, the genetic, biochemical, and functional effects prompted by two variants, each found in a non-familial SLNE or a DEE patient but both affecting nucleotides at the KCNQ2 intron 6-exon 7 boundary, have been investigated to test whether and how they affected the splicing process and to clarify whether such mechanism might play a pathogenetic role in these patients. Analysis of KCNQ2 mRNA splicing in patient-derived lymphoblasts revealed that the SLNE-causing intronic variant (c.928-1G > C) impeded the use of the natural splice site, but lead to a 10-aa Kv7.2 in frame deletion (Kv7.2 p.G310Δ10); by contrast, the DEE-causing exonic variant (c.928G > A) only had subtle effects on the splicing process at this site, thus leading to the synthesis of a full-length subunit carrying the G310S missense variant (Kv7.2 p.G310S). Patch-clamp recordings in transiently-transfected CHO cells and primary neurons revealed that both variants fully impeded Kv7.2 channel function, and exerted strong dominant-negative effects when co-expressed with Kv7.2 and/or Kv7.3 subunits. Notably, Kv7.2 p.G310S, but not Kv7.2 p.G310Δ10, currents were recovered upon overexpression of the PIP_2_-synthesizing enzyme PIP5K, and/or CaM; moreover, currents from heteromeric Kv7.2/Kv7.3 channels incorporating either Kv7.2 mutant subunits were differentially regulated by changes in PIP_2_ availability, with Kv7.2/Kv7.2 G310S/Kv7.3 currents showing a greater sensitivity to PIP_2_ depletion when compared to those from Kv7.2/Kv7.2 G310Δ10/Kv7.3 channels. Altogether, these results suggest that the two variants investigated differentially affected the splicing process at the intron 6-exon 7 boundary, and led to the synthesis of Kv7.2 subunits showing a differential sensitivity to PIP_2_ and CaM regulation; more studies are needed to clarify how such different functional properties contribute to the widely-divergent clinical phenotypes.

## Introduction

KCNQ2 and KCNQ3 genes encode for Kv7.2 and Kv7.3 voltage-gated K^+^ channel subunits each showing six transmembrane segments (S_1_-S_6_) and cytoplasmic N- and C-termini of variable length; the pore domain is encompassed by the S_5_-S_6_ segments and the intervening linker of each subunit, whereas the transmembrane segments between S_1_ and S_4_ form the voltage-sensing domain (VSD). The C-terminal region of Kv7 subunits contains domains (called from A to D) required for homo- or heteromeric subunit assembly and for a complex network of mutually interacting molecules, such as phosphatidylinositol 4,5-bisphosphate (PIP_2_), calmodulin, syntaxyn, A-kinase-anchoring proteins, which allow Kv7 channel regulation by pKC, and ankyrin-G (Soldovieri et al., 2011). Kv7.2/Kv7.3 channels are mainly expressed in neuronal cells, where they contribute to a slowly activating and deactivating K^+^ current called I_KM_ (or M-current) as it is inhibited by the activation of M_1_ receptors (Selyanko et al., 2000). The molecular pathways leading to M-current inhibition include pKC phosphorylation of the Kv7.2 subunits, dissociation of CaM from the channel and a consequent reduced affinity towards PIP_2_ (Kosenko et al., 2012).

I_KM_ controls neuronal excitability in the sub-threshold range for action potential generation (Marrion, 1997; Wang et al., 1998); thus, it is not surprising that pathogenic variants in KCNQ2 and KCNQ3 genes have been identified in epileptic disorders, ranging from Self-Limiting Familial Neonatal Epilepsy (SLFNE), characterized by seizures occurring in the first days of life and disappearing after few months, without significant effects on the subsequent neuropsychomotor development, to developmental and epileptic encephalopathies (DEEs), most often characterized by severe epileptic conditions, associated to variable degrees of social, cognitive, motor, language, and behavioral impairments, and pharmacoresistance (Weckhuysen et al., 2012; Weckhuysen et al., 2013). Although the causes for this differential developmental trajectories are still largely unknown, some genotype-phenotype correlations have emerged; in fact, while most variants causing SLFNE are nonsense, splice, or frameshifts randomly distributed throughout the gene, those causing DEE are almost invariably missense and cluster in four hot-spots channel domains exerting critical functional roles (Millichap et al., 2016).

Characterization of the specific effects prompted by each KCNQ2 variant on channel function could allow to identify distinct pathogenetic mechanisms, thus corroborating genotype-phenotype correlations between novel KCNQ2 variants and disease severity, with a potentially-relevant impact on the clinical management of these patients. Functional studies, mainly performed with missense variants, have revealed that, when compared with SLFNE-causing variants, associated with haploinsufficiency, most of the DEE-causing substitutions prompt strong loss-of-function (LoF) (Miceli et al., 2013), dominant-negative (Orhan et al., 2014) or gain-of-function (GoF) (Miceli et al., 2015; Millichap et al., 2017) effects. No study has yet been performed in KCNQ2 splice-site variants. Therefore, the genetic, biochemical, and functional effects prompted by two variants, each found in a SLNE (Soldovieri et al., 2014) or a DEE patient (Soldovieri et al., 2020) but both affecting nucleotides at the KCNQ2 intron 6-exon 7 boundary, have been investigated in the present study to test whether and how they affected the splicing process and to clarify whether such mechanism might play a pathogenetic role in the probands carrying these two variants.

## Materials and Methods

### Patients

Parents of each patient signed an informed consent for the participation, according to the French bioethics law. The clinical features of the two patients herein investigated have been previously described (Soldovieri et al., 2014; Soldovieri et al., 2020).

Briefly, the girl with early-onset DEE caused by the heterozygous NM_172107.4:c.928 G > A mutation described in Soldovieri et al. (2020) showed burst-suppression pattern at the EEG, recurrent seizures with multifocal onset occurring during the first days of life, and was refractory to different therapeutic trials with phenobarbital, phenytoin, levetiracetam, vigabatrin and pyridoxine. The neurologic evolution was characterized by severe psychomotor delay, absent speech, apostural tetraparesis, and strabismus. A significant decrease of the epileptiform activity was observed following introduction of gabapentin, a newly-identified Kv7 activator (Manville and Abbott, 2018) able to counteract *in vitro* mutation-induced LoF effects (Soldovieri et al., 2020).

The male patient carrying a heterozygous NM_172107.4:c.928-1G > C mutation described in Soldovieri et al. (2014; Individual II-I in family 9) had seizures in the neonatal period but no recurrence thereafter, normal EEG and normal neurodevelopment; the variant was not detected in either parent, and occurred *de novo*, thus confirming the diagnosis of non-familial Self-Limiting Neonatal Epilepsy (SLNE; Soldovieri et al., 2014).

### Genetic Analysis on Immortalized Lymphoblasts

For the c.928-1G > C variant, the validation of the effects on Kv7.2 mRNAs splicing was performed as previously described (Soldovieri et al., 2014). Briefly, mRNA was extracted from Epstein-Barr Virus-immortalized lymphoblastoid cells with the RNeasy minikit (Qiagen, Courtaboeuf, France). Retrotranscription was performed using the Expand RT kit (Invitrogen, Saint Aubin, France). PCR was performed with primers in exon 5 (forward: 5′-TAC​GCG​GAT​GCA​CTC​TGG-3′) and exon 8 (reverse: 5′-GTG​GAG​TGC​AGG​TCT​GTG​C-3′); PCR products were subsequently sequenced.

For the c.928G > A variant, RNA was extracted from Blood samples collected on PAXgene Blood Tube using the PAXgene Blood RNA Kit (Qiagen). cDNA was synthesized from RNA using the SuperScriptTM II RT (ThermoFisher Scientific) according to the manufacturer instructions with primers in exon 6 (forward: 5′-CCT​TGC​GGC​AAC​CTT​CAC-3′) and exon 8 (reverse: 5′- GCT​CGT​AGT​ACT​GCC​ACG-3′). cDNA amplicons were separated on a gel electrophoresis using a Labchip GX (Perkin Elmer). Both variants were submitted to ClinVar database (SCV001423824.1 and SCV000484597.1).

### Mutagenesis and Heterologous Expression of Channel Subunits

As reported (Soldovieri et al., 2020), c.928G > A/p.G310S variant was engineered in a pcDNA3.1-Kv7.2 plasmid for electrophysiological and western-blot experiments, as well as in a dual-tagged Enhanced Green Fluorescent Protein-Kv7.2-hemagglutinin (EGFP-Kv7.2-HA) plasmid used for experiments in neurons, by Quick-change mutagenesis (Agilent Technologies), accordingly to previously described procedure (Miceli et al., 2012). In the EGFP-Kv7.2-HA chimeric construct, in addition to an EGFP at the cytoplasmic N-terminus, an HA epitope was inserted in the extracellular loop that connects transmembrane domains S_1_ and S_2_ of Kv7.2 subunits (Schwake et al., 2000; Soldovieri et al., 2006). By contrast, the cDNA for the Kv7.2 p.G310Δ10 variant was engineered by Eurofins service (Eurofins Genomics, Germany); then, a BspeEI/BstXI restriction cassette encompassing variant position was extracted and inserted into the dual-tagged EGFP-Kv7.2-HA plasmid, previously reacted with the same enzymes. Successful cloning was checked by direct sequencing.

Methods for CHO cell growth and primary cortical neurons isolation, as well as protocols for wild-type and mutant cDNAs expression by transient transfection have been previously described (Soldovieri et al., 2016; Soldovieri et al., 2020). Total cDNA in the transfection mixture was kept constant at 4 μg (for electrophysiological recordings in CHO cells), 2 μg (for electrophysiological recordings in neurons), or 6 μg (for Western-blotting experiments).

### Cell Surface Biotinylation and Western-Blot

Expression of wild-type or variant Kv7.2 subunits in CHO cells in total lysate or plasma membrane-enriched fractions was investigated by surface biotinylation and western-blotting analysis, as described (Soldovieri et al., 2014). Channel subunits were identified using mouse monoclonal anti-Kv7.2 primary antibodies (clone N26A/23, dilution 1:1,000; Antibodies Inc., Davis, CA), followed by horseradish peroxidase (HRP)-conjugated anti-mouse secondary antibodies (clone NA931V; dilution 1:5,000; GE Healthcare, Little Chalfont, United Kingdom).

### Patch-Clamp Recordings

Macroscopic current recordings were performed in CHO cells (24 h after transfection) or in neurons (48 h after transfection), as previously reported (Miceli et al., 2013; Binda et al., 2018). Current densities were obtained dividing the current amplitude measured at each test voltage by the cell capacitance (pA/pF). The conductance was calculated by normalizing the currents measured at the end of each pulse to the driving force. Experimental data were fitted to a Boltzmann equation of the following form: *y*=max/[1+exp(*V*_1/2_−*V*)/*k*] to obtain the voltage of half activation (V_½_) and the slope factor (*k*).

Experiments on primary cortical neurons were performed either in voltage-clamp mode, using protocols similar to those applied on CHO cells, or in current-clamp mode, to record membrane resting potential. Data were acquired with a Multiclamp 700B amplifier and Digidata 1440A (Axon Instruments, Molecular Device) and pClamp 10.3 software (Molecular Devices) and analyzed with Clampfit 10.3 software (Molecular Devices).

Experiments with the Danio rerio voltage-sensing phosphatase (DrVSP) were performed as previously reported (Ambrosino et al., 2018). Briefly, a 0 mV step lasting 1.5 s was used to activate Kv7 currents, followed by a +100 mV step of variable length (from 0.1 to 1 s, with increments of 0.1 s) to activate DrVSP, and by a second 0 mV step, causing DrVSP switch off and Kv7 current recovery (25 s). Time-dependent current decline following DrVSP activation was quantified as the ratio between the current size measured at the beginning of the second 0 mV step (after DrVSP activation) versus that measured at the end of the first 0 mV step (before DrVSP activation), expressed as a function of the duration of the +100 mV step; these values were then fitted to a Boltzmann function of the following form: y = 1/[1+(x/t_½_)-Hill slope], where t_½_ was the duration of the +100 mV step required to inhibit 50% of maximal currents. Kv7 current recovery was instead quantified by normalizing current values recorded during the entire second step at 0 mV to the currents measured at the end of the first 0 mV step. The kinetics of DrVSP-induced Kv7 current inhibition and of Kv7 current recovery after DrVSP switch off were measured by fitting the current traces to a single exponential function of the following form: y = amp×e(−t/τ)+c, where τ was the time constant of the function.

### Statistics

Each data point shown in figures or in the text is the Mean ± SEM of at least 4 determinations, each performed in a single cell or in a separate experiment. Statistically significant differences were evaluated with the Student’s t-test or with the ANOVA followed by the Student-Newman-Keuls test, with the threshold set at *p* < 0.05.

## Results

### Analysis of Kv7.2 mRNAs Expressed in Patient-Derived Lymphoblasts

**FIGURE 1:**
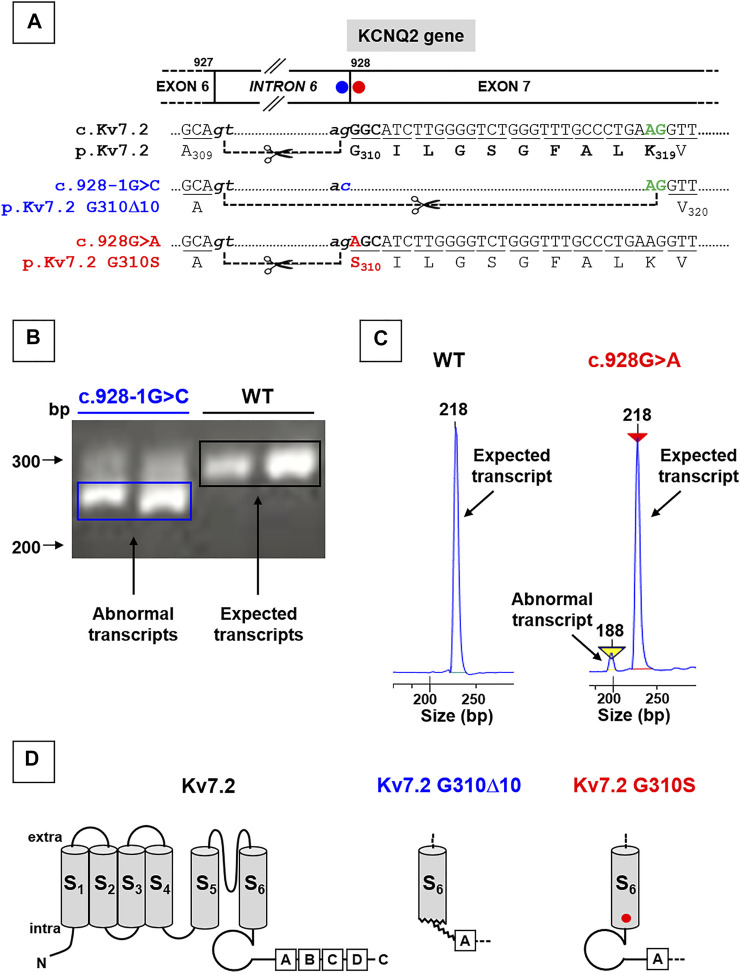
Localization of KCNQ2 variants herein investigated and effects on KCNQ2 mRNAs expression. **(A)** Localization of the two variants herein investigated in the KCNQ2 gene, falling at the last nucleotide of intron 6 (KCNQ2 **(C)**928-1G > C; blue circle; already reported in Soldovieri et al., 2014) or at the first nucleotide of exon 7 (KCNQ2 **(C)**928G > **(A)**; already reported in Soldovieri et al., 2020). **(B)** Representative agarose gel run and **(C)** electrophoresis profiles of major bands obtained from RT-PCR experiments performed on Kv7.2 mRNAs expressed in father- or probands-deriving lymphoblasts, as indicated; the mRNA fragment of 295 bp **(B)** or 218 bp **(C)** are expected when the ag acceptor site at the end of intron 6 is used, while those of 265 bp **(B)** or 188 bp **(C)** bands are obtained using the alternative splice site in the exon 7. Panels in C have been obtained cropping the regions of interest from the complete electrophoresis profiles, reported in Supplementary Figure S2 Topology of a Kv7.2 subunit showing the 6 transmembrane segments (called from S1 to S6) and four intracellular domains at the C-terminus (called from A to D), binding many regulatory molecules. Middle and left panels show the structural alterations prompted by the Kv7.2 G310Δ10 in-frame deletion or by the missense Kv7.2 G310S variant, respectively.

Both variants herein reported fall at the intron 6-exon 7 boundary of the KCNQ2 gene (Figure 1A). Four in silico tools provided by Alamut software (SpliceSiteFinder-like, MaxEntScan, NNSPLICE, and Gene Splicer; Suppl. Figure 1A) revealed that the SLNE-causing c.Kv7.2 c.928-1G > C intronic variant (affecting the g of the ag acceptor site located at the end of intron 6; Figure 1A) drastically reduced the strength of the affected splice site (Supplementary Figure S1A; Soldovieri et al., 2014); similar results were obtained with the software spliceAI (acceptor loss score: 0.96). In keeping with this prediction, while Kv7.2 mRNAs analysis in lymphoblasts from the unaffected, non-carrier father revealed the presence of only wild-type fragments (295 bp), transcript analysis in lymphoblasts from the proband carrying the c.928-1G > C variant revealed the presence of both wild-type (295 bp) and shorter (265 bp) fragments (Figure 1B), the latter corresponding to the variant-induced use of a cryptic ag site located 30 bp downstream in exon 7, thus causing the removal of encompassed nucleotides (Figure 1A). The intensity of this low molecular weight band was much stronger than that corresponding to the full-length band of the wild-type allele, leading to an intensity ratio higher than the 1:1 expected from the heterozygous state of the patient; this result, possibly explained by the more efficient amplification of shorter amplicons, suggests that the effect of the variant on the splicing is complete. Expression of shorter mRNAs causes a 10 aminoacid in-frame deletion (Kv7.2 p.G310_K319del10, herein referred as Kv7.2 p.G310Δ10), affecting the end of S6 domain and the beginning of the C-terminus of Kv7.2 subunits (Figure 1D).

By contrast, in the case of the DEE-causing exonic c.928G > A variant, affecting the first nucleotide of exon 7 (Figure 1A), prediction tools provided by Alamut (SpliceSiteFinder-like, MaxEntScan, NNSPLICE, and Gene Splicer) were in favor of only a slight reduction of the strength of the splice site (Supplementary Figure S1A); similar results were obtained with the software spliceAI (acceptor loss score: 0.06). Accordingly, PCR analysis performed in lymphoblasts from the patient carrying this variant revealed that, while only the expected 218 bp-long fragments were detected in samples from the father (used as a control), a shorter (188 bp) Kv7.2 mRNA fragment was also detected in the patient carrying the c.928G > A variant (Figure 1C), with the first being much more abundant than the latter (90:10, respectively) (Figure 1C). The most abundant mRNA incorporates the c.928G > A substitution in exon 7, causing the missense substitution p.G310S (Figures 1A–D).

### Functional Characterization of Kv7.2 Channels Carrying p.G310S or p.G310Δ10 Variants in CHO Cells

**FIGURE 2:**
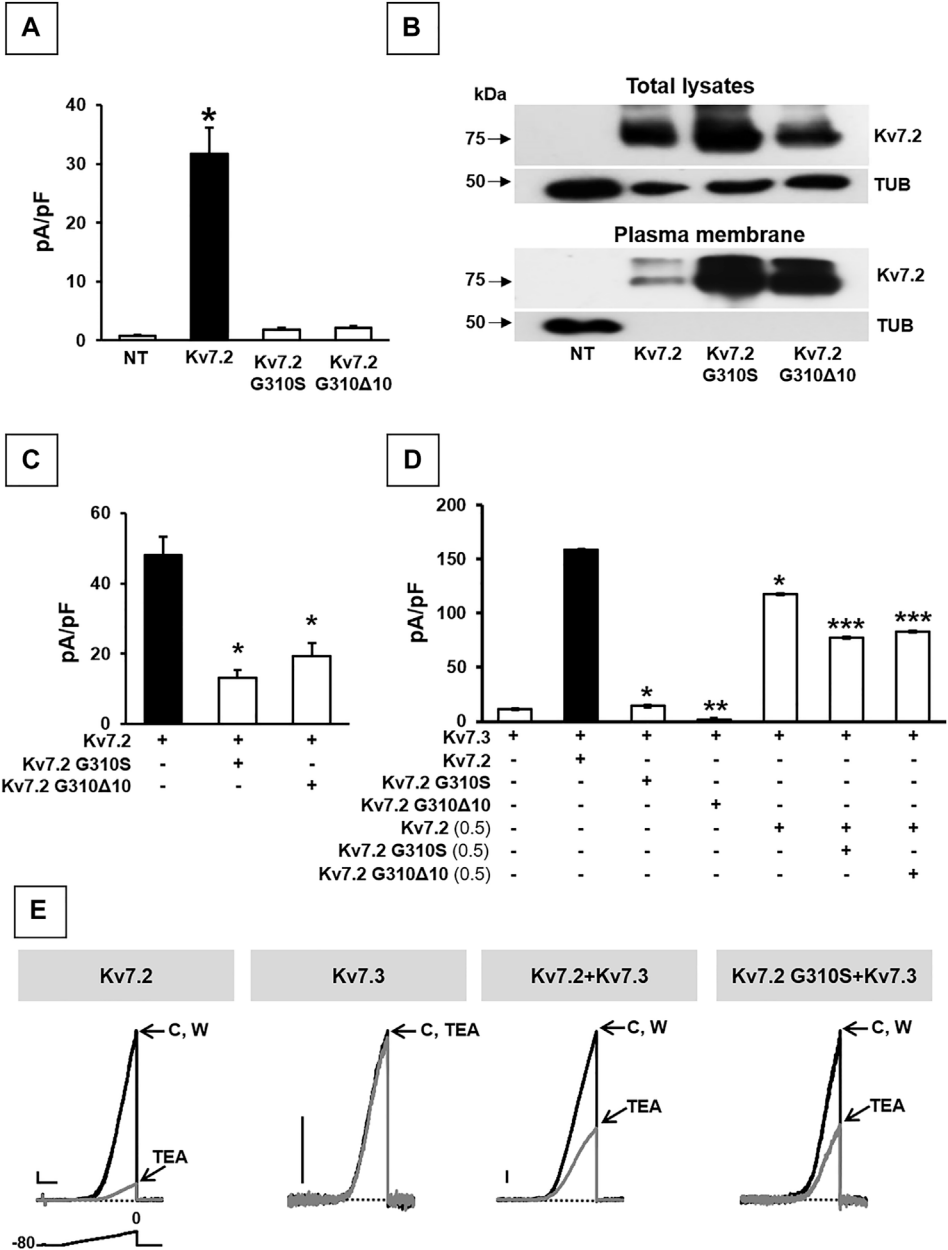
Functional and biochemical characterization of Kv7.2 G310S and Kv7.2 G310Δ10 subunits. **(A)** Maximal current densities measured in non transfected cells (NT) or in cells expressing wild-type or mutant Kv7.2 subunits, as indicated. * = *p* < 0.05 versus NT cells. **(B)** Western blot analysis of proteins from total lysates (upper panels) or biotinylated plasma membrane fractions (lower panels) from CHO cells transfected with the indicated constructs. Higher and lower blots were probed with anti-Kv7.2 or anti-α-tubulin antibodies, as indicated. Numbers on the left correspond to the molecular masses of the protein markers. ODKv7.2 TOT/ODTUB ratios were 1.00 ± 0.20, 1.54 ± 0.19, or 0.66 ± 0.25; n = 4; *p* > 0.05 among each other; ODKv7.2 BIOT/ODTUB ratios were 1.00 ± 0.50, 5.2 ± 1.50, or 3.33 ± 0.45 in Kv7.2-, Kv7.2 p.G310S-, or Kv7.2 p.G310Δ10-transfected cells, respectively; n = 4; *p* < 0.05 versus Kv7.2. **(C,D)** Quantification of maximal currents measured in CHO cells expressing either Kv7.2 variant with Kv7.2 **(C)** and/or Kv7.3 **(D)** subunits, as indicated. * = *p* < 0.05 versus the black bar in each panel; ** = *p* < 0.05 versus Kv7.3; *** = *p* < 0.05 Kv7.2 + Kv7.3 (0.5:1). **(E)** Representative current traces recorded upon application of the ramp protocol (shown below the first traces) on CHO cells expressing the indicated constructs in control solution **(C)**, after 2-min 3 mM TEA perfusion, or upon drug washout (W). Current scale: 100 pA; time scale: 1 s.

The functional consequences of p.G310Δ10 or p.G310S Kv7.2 subunits were investigated by electrophysiological recordings upon their expression in CHO cells by transient transfection. Expression of Kv7.2 channels in these cells elicited robust voltage-gated outward K^+^ currents with slow activation and deactivation kinetics, lack of inactivation and activating at about −40 mV, with a current density of about 30 pA/pF (Figure 2A; Table 1). By contrast, K^+^ current densities recorded from CHO cells transfected with either mutant plasmids were identical to those of non-transfected cells (Figure 2A; Table 1).

**TABLE 1:** Functional properties of currents of wild-type or mutant Kv7.2 subunits when expressed in CHO cells or cortical neurons.

CHO CELLS	Control solution				3 mM TEA	
Experimental group in CHO cells (cDNA transfection ratio)	n	pA/pF (@0 mV)	V_½_ (mV)	*k* (mV/efold)	n	TEA-sensitive current (%)
NT	4	0.8±0.1^*^	-	-	-	-
Kv7.2^##^	10	31.7±4.4	−25.2±0.8	10.4±0.7	4	89.8±1.6
Kv7.2 G310S^##^	10	1.9±0.2^*^	-	-	-	-
Kv7.2 G310Δ10	7	2.1±0.4^*^	-	-	-	-
Kv7.2 + Kv7.2 G310S (1:1)	21	13.2±2.3^*^	−24.5±0.8	8.7±0.7	-	-
Kv7.2 + Kv7.2 G310Δ10 (1:1)	19	19.3±3.6^*^	−17.4±0.8^*,&^	11.8±0.6^&^	-	-
Kv7.3^##^	12	11.2±1.5	−38.5±0.5	5.3±0.4	4	6.7±1.8^*^
Kv7.2 + Kv7.3 (1:1)^##^	15	158.5±12.7^*,**^	−26.2±0.5	9.9±0.4	4	45.0±2.6^*,**^
Kv7.2 + Kv7.3 (0.5:1)^##^	21	117.5±9.1^#^	−29.8±0.5^#^	9.7±0.4	-	-
Kv7.2 G310S + Kv7.3 (1:1)^##^	14	14.3±2.1^#^	−26.1±0.9^**,$^	9.9±0.8	8	47.2±3.9^*,**^
Kv7.2 G310Δ10 + Kv7.3 (1:1)	8	1.9±0.3^**,#,†^	-	-	-	-
Kv7.2 + Kv7.2 G310S + Kv7.3 (0.5:0.5:1)^##^	24	77.2±7.8^#,$^	-26.4±0.4^$^	9.0±0.3^#^	10	46.6±3.6^*,**^
Kv7.2 + Kv7.2 G310Δ10+Kv7.3 (0.5:0.5:1)	25	82.9±7.8^#,$^	−27.8±0.4^#,$,β^	8.5±0.3^#,$^	4	55.6±11.5^*,**^
**Experimental group in neuronal cells (cDNA transfection ratio)**	**n**	**pA/pF (@40 mV)**	**V_½_ (mV)**	***k* (mV/efold)**	**n**	**V_REST_**
NT^†^	38	48.8±8.7	−6.8±9.9	15.0±0.8	38	−41.9±2.4
Kv7.2+Kv7.3 (1:1)^##^	18	157.2±68.9^§§^	−34.4±9.7	15.1±4.8	18	−52.8±1.7^§,§§^
Kv7.2 G310S + Kv7.3 (1:1)^##^	22	39.0±16.9^#^	−35.6±4.6	16.3±5.5	22	−37.9±3.0
Kv7.2 G310Δ10 + Kv7.3 (1:1)	12	26.9±7.6^#^	−1.2±2.1^#^	17.2±1.9	12	−26.9±3.8^#,§,§§^
Kv7.2 + Kv7.2 G310S + Kv7.3 (0.5:0.5:1)^##^	24	74.2±26.3	−26.8±9.1	14.2±3.6	22	−42.5±4.0
Kv7.2 + Kv7.2 G310Δ10 + Kv7.3 (0.5:0.5:1)	13	64.1±22.6	−26.7±7.1	18.3±1.3	13	−41.5±4.4

* = *p* < 0.05 vs. Kv7.2; ** = *p* < 0.05 vs. Kv7.3; ^&^ = *p* < 0.05 vs. Kv7.2 + Kv7.2 G310S.; ^#^ = *p* < 0.05 vs. Kv7.2 + Kv7.3 (1:1); ^$^ = *p* < 0.05 vs. Kv7.2 + Kv7.3 (0.5:1); ^†^ = *p* < 0.05 vs. Kv7.2 G310S + Kv7.3(1:1).; ^β^ = *p* < 0.05 vs. Kv7.2 + Kv7.2 G310S + Kv7.3(0.5:0.5:1); ^§^ = *p* < 0.05 vs. non transfected neurons.; ^§§^= *p* < 0.05 vs. all other experimental groups in neurons.

^##^Values already reported in Soldovieri et al. (2020).

To investigate possible variant-induced changes in Kv7.2 subunits trafficking to the plasma membrane, Western-blot experiments were performed in both total lysates and plasma membrane-enriched fractions from CHO cells expressing Kv7.2, Kv7.2 G310S, or Kv7.2 G310Δ10 subunits. The results obtained showed that either substitutions did not interfere with whole-cell expression of Kv7.2 subunits (Figure 2B; *p* > 0.05 among each other), whilst both significantly increased Kv7.2 subunit expression at the plasma membrane (Figure 2B; *p* < 0.05 versus Kv7.2). Although this increase in plasma membrane abundance, quantitatively similar for both variants, was not further investigated, these results suggested that the functional impairment observed in electrophysiological experiments upon transfection with plasmids carrying either variant was not due to the inability of Kv7.2 mutant subunits to efficiently traffic to the plasma membrane.

Having detected non-functional mutant subunits at the plasma membrane, we also investigated whether they interfered with the function of wild-type Kv7.2 and/or Kv7.2 subunits, as Kv7.2/3 heteromers underlie most of the M-current in adult neurons (Wang et al., 1998). Notably, co-expression of Kv7.2 subunits carrying either variant with Kv7.2 or Kv7.3 + Kv7.3 subunits significantly reduced maximal currents, thus indicating mutation-induced dominant-negative effects on Kv7.2 (Figure 2C; Table 1) or Kv7.2/Kv7.3 (Figure 2D; Table 1) currents. By contrast, significant dominant-negative effects on Kv7.3 currents only occurred upon co-expression of Kv7.2 p.G310Δ10, but not Kv7.2 p.G310S subunits, whereas currents levels in Kv7.3- or Kv7.2 G310S/Kv7.3-expressing cells were identical (Figure 2D; Table 1). To discern whether currents measured in Kv7.2 G310S/Kv7.3-expressing cells were carried by homomeric Kv7.3 or Kv7.2 G310S/Kv7.3 heteromeric channels, pharmacological experiments using tetraethylammonium (TEA, 3 mM) were performed; in fact, TEA is widely used to discriminate among Kv7 channel of distinct subunit composition since it blocks homomeric Kv7.2, homomeric Kv7.3 or heteromeric Kv7.2/Kv7.3 channels with a high, low, or intermediate potency, respectively (Hadley et al., 2000). In these experiments, currents from cells expressing Kv7.2 G310S/Kv7.3 channels were blocked by TEA to a similar extent as Kv7.2/Kv7.3 channels (Figure 2E; Table 1), thus suggesting that Kv7.2 p.G310S subunits form functional heteromeric channels with Kv7.3 subunits.

### Functional Characterization of Kv7.2 Channels Carrying p.G310S or p.G310Δ10 Variants in Cortical Neurons

**FIGURE 3:**
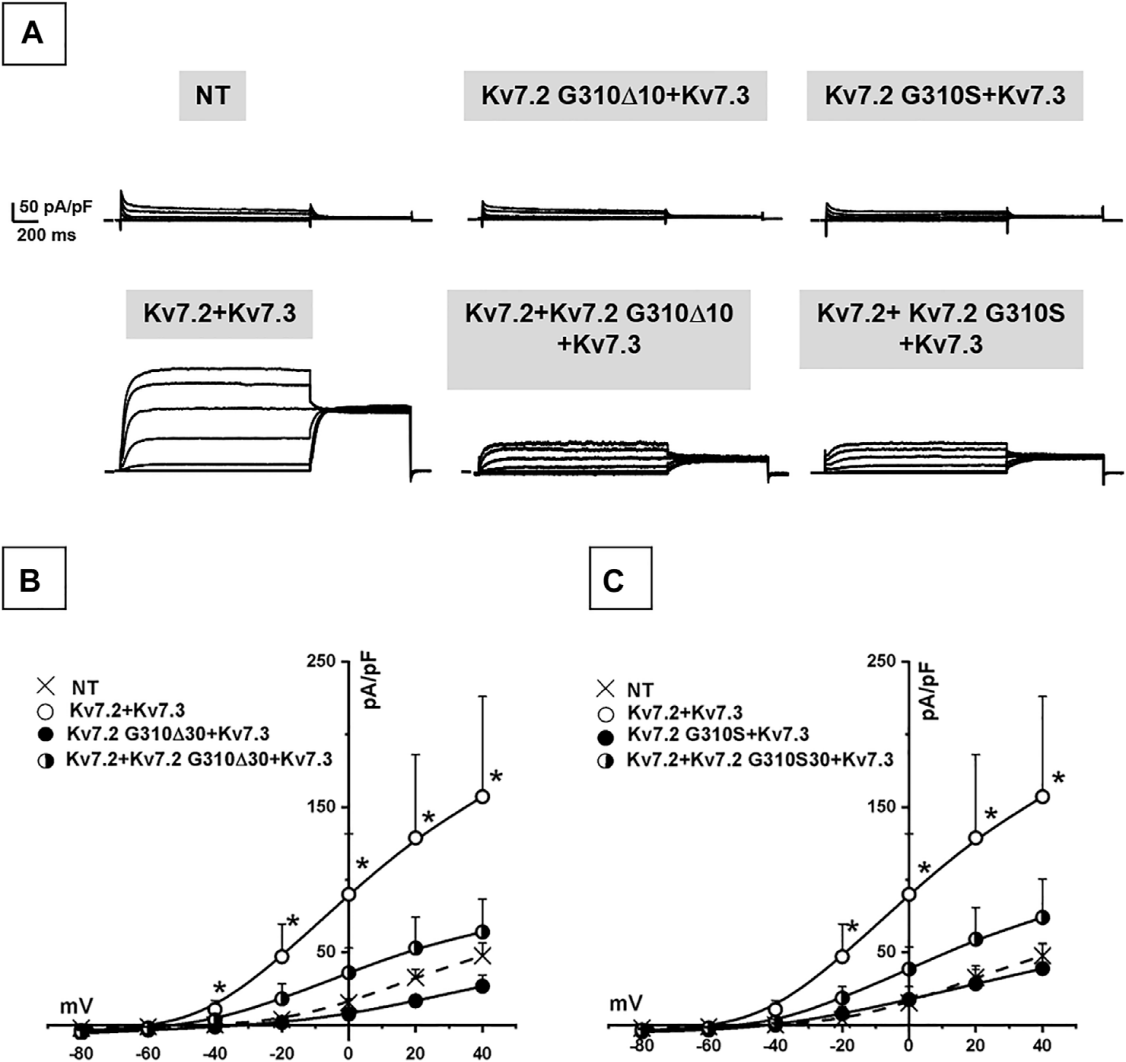
Functional characterization of heteromeric Kv7.2 + Kv7.3 channels incorporating Kv7.2 G310S or Kv7.2 G310Δ10 subunits expressed in neurons. **(A)** Macroscopic currents from neurons expressing wild-type or mutant Kv7.2 + Kv7.3 subunits, as indicated. **(B,C)** Current density/voltage curves measured in non transfected neurons (NT) or expressing the indicated channels incorporating Kv7.2 G310Δ10 **(B)** or Kv7.2 G310S **(C)** subunits, as indicated.

Co-expression of Kv7.2 + Kv7.3 subunits in cortical neurons yielded robust outward currents with gating properties very similar to those measured in CHO cells (Figure 3A; Table 1). Notably, the co-expression of Kv7.2 p.G310S or Kv7.2 p.G310Δ10 subunits with wild-type Kv7.3 subunits reduced current densities to background (non-transfected) levels (Table 1). Moreover, cells co-expressing either Kv7.2 variant subunits with Kv7.2 + Kv7.3 subunits channels carried currents whose size was roughly intermediate between that of non-transfected neurons and those expressing Kv7.2 + Kv7.3 channels (Figures 3B,C; Table 1). When compared to non-transfected neurons, neuronal resting membrane potential was hyperpolarized upon Kv7.2 + Kv7.3 co-expression; by contrast, co-expression of either variant failed to cause membrane hyperpolarization (Table 1). Altogether, these results validate the results obtained in CHO cells and suggest that both p.G310Δ10 and p.G310S Kv7.2 variants strongly suppress Kv7.2 and/or Kv7.3 channel function.

### p.G310S and p.G310Δ10 Kv7.2 Subunits Show Distinct Changes in PIP_2_/CaM Regulation

Both Kv7.2 variants herein investigated affect bottom end of the S_6_ segment in the pore of Kv7.2 subunits (Figure 1C); this region is critical for PIP_2_-dependent regulation of Kv7.2 subunit function (Zhang et al., 2003; Telezhkin et al., 2013; Zhang et al., 2013; Soldovieri et al., 2016), and is located just prior helix A in the C-terminus (Figure 1C), a major anchoring site for CaM (Yus-Najera et al., 2002; Gamper and Shapiro, 2003).

Both PIP_2_ and CaM positively affect Kv7.2 subunit function (Telezhkin et al., 2013; Ambrosino et al., 2015). To investigate whether the loss-of-function effects caused by either Kv7.2 variant were dependent on changes in PIP_2_- and/or CaM-dependent modulation, patch-clamp recordings were performed in CHO cells in which PIP_2_ levels were either increased by the co-expression of the PIP_2_-synthesizing enzyme PIP5K (Wenk et al., 2001; Winks et al., 2005), or decreased by a voltage-dependent phosphatase (DrVSP) (Falkenburger et al., 2010); in addition, CaM regulation was tested by measuring the functional consequences of an enhanced CaM availability, achieved by co-expressing wild-type CaM or CaM_1234_, a CaM variant unable to bind Ca2+ (Geiser et al., 1991).

**FIGURE 4:**
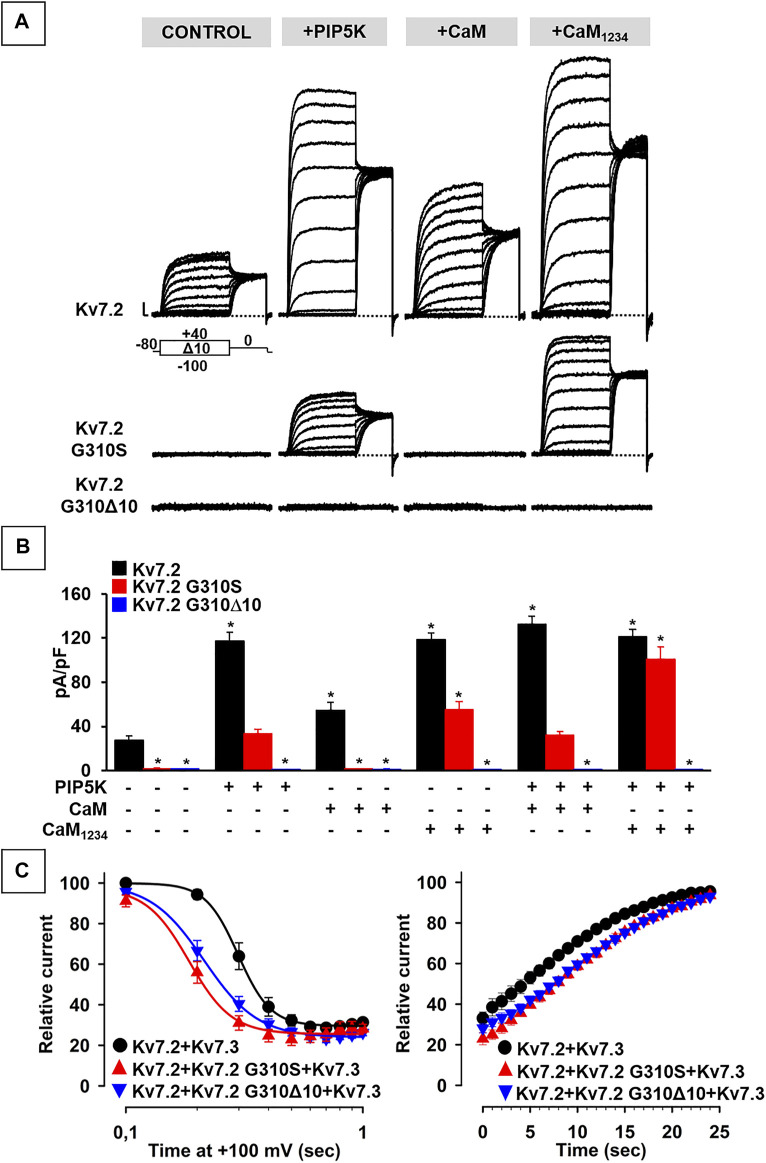
Functional study of the modulation of Kv7.2 G310S or Kv7.2 G310Δ10 subunits by PIP_2_ and calmodulin. **(A,B)** Representative traces **(A)** and quantification **(B)** of currents measured in CHO cells co-expressing homomeric wild-type or mutant Kv7.2 subunits with PIP5K, CaM, and/or CaM_1234_, as indicated. * = *p* < 0.05 versus Kv7.2. **(C)** Time-dependence of current decrease (left panel) and recovery (right panel) of currents measured in CHO cells co-expressing the indicated channels with DrVSP.

The results obtained suggest that Kv7.2 currents were significantly increased upon co-expression of PIP5K, CaM, or CaM_1234_ (Figure 4A; Table 2); notably, potentiation by PIP5K, CaM, or CaM_1234_ was non-additive in the present experimental conditions. Instead, Kv7.2 p.G310S channels showed strong functional recovery in the presence of PIP5K and CaM_1234_, but not wild-type CaM (Figure 4A; Table 2); notably, the effects of PIP5K and CaM_1234_ were synergistic (Figure 4B; Table 1). By contrast, none of the previously mentioned experimental condition (alone or in combination) was able to recover Kv7.2 p.G310Δ10 function (Figures 4A,B; Table 1), possibly suggesting that the G310-K319 region deleted by the mutation is critically involved in Kv7.2 currents functional modulation by PIP_2_ and/or CaM.

**TABLE 2:** Functional properties of currents measured in CHO cells expressing wild-type or mutant Kv7.2 subunits upon modulation of PIP_2_ or CaM levels.

Experimental group (cDNA transfection ratio)	n	pA/pF (0 mV)	V_½_ (mV)	*k* (mV/efold)
Kv7.2 + pcDNA3 (1:8)	10	27.6±3.6	−26.8±0.6	10.1±0.5
Kv7.2 G310S + pcDNA3 (1:8)	10	1.9±0.2^*^	-	-
Kv7.2 G310Δ10 + pcDNA3 (1:8)	10	1.4±0.1^*^	-	-
Kv7.2+PIP5K + pcDNA3 (1:3:5)	23	117.1±8.2^*^	−42.2±0.7^*^	9.8±0.4
Kv7.2 G310S + PIP5K + pcDNA3 (1:3:5)	17	33.5±4.3^**^	−30.6±0.7^*,**^	9.7±0.6
Kv7.2 G310Δ10 + PIP5K + pcDNA3 (1:3:5)	12	1.1±0.1^*,**,***^	-	-
Kv7.2 + pcDNA3 + CaM (1:3:5)	20	54.7±7.1^*^	−25.3±0.5	9.4±0.4
Kv7.2 G310S + pcDNA3 + CaM (1:3:5)	11	1.5±0.5^*^	-	-
Kv7.2 G310Δ10 + pcDNA3 + CaM (1:3:5)	8	1.2±0.3^*^	-	-
Kv7.2 + pcDNA3 + CaM_1234_ (1:3:5)	36	118.5±4.5^*^	−37.2±0.7^*^	11.7±0.5
Kv7.2 G310S + pcDNA3 + CaM_1234_ (1:3:5)	20	54.9±7.6^*^	−26.1±0.6	11.3±0.5
Kv7.2 G310Δ10 + pcDNA3 + CaM_1234_ (1:3:5)	12	1.1±6.6^*^	-	-
Kv7.2 + PIP5K + CaM (1:3:5)	27	132.8±7.3^#^	−40.6±0.5^#^	10.6±0.5
Kv7.2 G310S+PIP5K + CaM (1:3:5)	22	32.1±3.5^##^	−30.4±0.6	8.8±0.5
Kv7.2 G310Δ10+PIP5K + CaM (1:3:5)	8	1.1±0.2^*^	-	-
Kv7.2 + PIP5K + CaM_1234_ (1:3:5)	13	121.6±6.2^*,**^	−43.7±0.9^*^	10.5±0.8
Kv7.2 G310S + PIP5K + CaM_1234_ (1:3:5)	20	100.8±11.1^***,&^	-36.1±0.7^***,&^	10.3±0.6
Kv7.2 G310Δ10 + PIP5K + CaM_1234_ (1:3:5)	12	1.0±0.1^*^	-	-

^*^ = p<0.05 vs Kv7.2+pcDNA3 (1:8); **=p < 0.05 vs Kv7.2 + PIP5K + pcDNA3 (1:3:5); *** = p < 0.05 vs Kv7.2 G310S + PIP5K + pcDNA3 (1:3:5);^#^ = p<0.05 vs Kv7.2 + pcDNA3 + CaM (1:3:5); ^##^ = p<0.05 vs Kv7.2 G310S + pcDNA3+CaM (1:3:5); ^&^ = p<0.05 vs Kv7.2 G310S + pcDNA3 + CaM_1234_ (1:3:5).

To further investigate variant-induced changes in PIP_2_-dependent Kv7.2 currents modulation, PIP_2_ levels were decreased upon co-expression of the voltage-dependent phosphatase DrVSP. As reported in Figure 4C, Kv7.2 + Kv7.3 currents were time-dependently inhibited when +100 mV depolarizing pulses lasting >0.2 s were used to activate DrVSP (t_½_ was 0.298 ± 0.003 s, n = 16), with a time constant of current inhibition (*τ*) of 0.24 ± 0.03 s (n = 13). Membrane repolarization at 0 mV, a membrane potential value at which the DrVSP is switched off, led to a complete time-dependent recovery of Kv7.2 + Kv7.3 currents with a time constant of 26.8 ± 3.27 s (n = 15). Notably, currents expressed by Kv7.2 + Kv7.2 G310S + Kv7.3 or Kv7.2 + Kv7.2 G310Δ10 + Kv7.3 channels were more easily inhibited upon DrVSP activation: in fact, t_½_ were 0.185 ± 0.008 or 0.214 ± 0.003 s, respectively (n = 12–15; *p* < 0.05 versus control and among each other; Figure 4C); in addition, a faster time constant was measured in cells expressing Kv7.2 + Kv7.2 G310S + Kv7.3 (τ = 0.15 ± 0.02 s; n = 9; *p* < 0.05 versus wild-type channels), but not Kv7.2 + Kv7.2 G310Δ10 + Kv7.3 subunits (τ = 0.24 ± 0.02 s; n = 10; *p* > 0.05 versus wild-type channels). No difference in time constant of current recovery was measured for Kv7.2 + Kv7.2 G310S + Kv7.3 or Kv7.2 + Kv7.2 G310Δ10 + Kv7.3 channels when compared to wild-type channels (τ = 26.5 ± 3.85 or 33.1 ± 4.77 s, respectively; n = 9–10; *p* > 0.05 versus wild-type channels).

Altogether, these results suggest that both variants herein studied prompted significant alterations in CaM- and PIP_2_-dependent Kv7.2 current modulation, with a greater functional derangement in the presence of the DEE-causing p.G310S variant when compared to the SLNE-causing p.G310Δ10 variant.

## Discussion

Pathogenic variants in Kv7.2 potassium channels have been found in patients affected by widely diverging epileptic phenotypes, ranging from self-limiting (SLFNE) to very severe DEEs (Weckhuysen et al., 2012; Weckhuysen et al., 2013). Given the rarity of KCNQ2-related disorders, genotype-phenotype correlations are difficult to establish; in general, haploinsufficiency caused by the loss of function of a single KCNQ2 allele (by nonsense, splice, or frameshift variants) is the most common cause of familial KCNQ2-SLFNE, while pathogenic variants so far identified in KCNQ2-DEE are all *de novo* missense variants, and are thought to exert more severe functional defects on potassium current function (Miceli et al., 2013; Orhan et al., 2014). No studies have yet been performed on KCNQ2 variants affecting splice sites (www.rikee.org).

In the present study, we have characterized the genetic, biochemical, and functional consequences prompted by two known variants in the KCNQ2 gene falling at the intron 6-exon 7 boundary: the SLNE-causing c.Kv7.2 c.928-1G > C intronic variant, and the DEE-causing exonic c.928G > A variant, to test whether and how they affected the splicing process and to clarify whether such mechanism might play a pathogenetic role in the probands carrying these two variants.

Kv7.2 mRNAs analysis from patient-derived immortalized lymphoblasts revealed that the SLNE-causing Kv7.2 c.928-1G > C substitution abolished the physiological “ag” acceptor site, and promoted the use of a cryptic site located 30 bp downstream, thus leading to a 10 aminoacid in-frame deletion (p.G310_K319Δ10); in the proband samples, comparison of the intensity of the new, mutation-induced, lower molecular weight band with that corresponding to the wild-type full-length band suggested that the mutation-induced effect of the variant on the splicing process is complete. By contrast, in the case of the c.928G > A substitution, mRNAs analysis revealed more complex effects, with most of the mRNAs (>90%) resulting from the use of the natural “ag” acceptor site localized at the end of intron 6, and leading to Kv7.2 mRNAs of the same length as wild-type, but encoding for the missense substitution p.G310S; in addition, the same splicing alteration described above and leading to the 10 aminoacid in-frame deletion (p.Gly310_Lys319Δ10) was also detected in this patient, although this component only accounted for <10% of total Kv7.2 mRNAs. Although splicing in lymphoblasts might not fully recapitulate that occurring at neuronal level, based on these results we have investigated the functional consequences of Kv7.2 p.G310Δ10 subunits produced by the intronic Kv7.2 c.928-1G > C variant, and those of Kv7.2 p.G310S subunits occurring as a result of the Kv7.2 c.928G > A exonic mutation.

Both Kv7.2 p.G310S and Kv7.2 p.G310Δ10 mutant subunits failed to elicit measurable currents and prompted dominant-negative effects in heteromeric configuration with Kv7.2 and/or Kv7.3 subunits when transiently-transfected in CHO cells. A similar lack of function was also observed when these subunits were co-expressed with Kv7.2/Kv7.3 subunits in primary culture of neonatal rat cortical neurons, a more physiological cellular context.

Kv7.2 currents are regulated by a complex network of mutually-interacting intracellular molecules (Hernandez et al., 2008); mutations herein investigated are located slightly upstream of the C-terminal helix A, a critical anchoring site for CaM (Yus-Najera et al., 2002; Gamper and Shapiro, 2003), and of one of the binding sites for the Kv7 activator PIP_2_ (Zhang et al., 2003; Li et al., 2005; Gamper and Shapiro, 2007; Telezhkin et al., 2013; Zhang et al., 2013; Soldovieri et al., 2016). Both CaM and PIP_2_ potentiate Kv7.2 currents; thus, the possibility existed that the observed mutation-induced LoF effects were dependent on alterations in the PIP_2_/CaM-dependent Kv7.2 current regulation. To test this hypothesis, the intracellular levels of these mediators were modified by overexpressing CaM (either in a wild-type or unable to bind Ca^2+^ isoform; Geiser et al., 1991), the PIP_2_-synthesizing enzyme PIP5K (thus increasing PIP_2_ levels; Wenk et al., 2001) or the PIP_2_-degrading voltage-dependent phosphatase from Danio rerio (Falkenburger et al., 2010).

In most of these experimental conditions (overexpression of PIP5K and/or CaM_1234_), currents carried by Kv7.2 subunits carrying the DEE-causing variant p.G310S were recovered to levels identical to those of wild-type Kv7.2 channels, suggesting that the LoF effects prompted by this variant on Kv7.2 currents are dependent to an alteration in the affinity of both mediators. By contrast, none of these experimental conditions (alone or in combination) were able to recover channel function in the case of Kv7.2 subunits incorporating the SLNE-causing variant p.G310Δ10, possibly because of a complete inability of CaM and/or PIP_2_ to bind Kv7.2 channels as a consequence of the variant-induced in-frame deletion. This could lead to a reduced incorporation of Kv7.2 subunits carrying the SLNE-causing variant in functional heterotetrameric channels and therefore in a reduction of dominant-negative effects prompted on Kv7.2/Kv7.3 currents at high CaM and/or PIP_2_ levels, thus providing a plausible molecular explanation for the more benign clinical phenotypes observed in the individual carrying this variant. Moreover, both Kv7.2/Kv7.2 G310S/Kv7.3 and Kv7.2/Kv7.2 G310Δ10/Kv7.3 currents were more sensitive than wild-type Kv7.2/Kv7.3 channels to PIP_2_ depletion; notably, the p.G310S variant prompted slightly stronger functional derangement when compared to the p.G310Δ10 variant, which could further contribute to the divergent clinical phenotypes observed in the two probands.

These results also suggest that a quantitative threshold level exists in terms of tolerance of Kv7.2 to variant-dependent changes in PIP_2_-dependent current regulation, with smaller effects (such those of the Kv7.2 p.G310Δ10 variant) leading to more benign forms of the clinical spectrum, and stronger derangements (as those caused by the Kv7.2 p.G310S variant) to more severe phenotypes. A similar hypothesis seems to provide a reasonable explanation for the results obtained in a DEE-affected patient carrying two variants in compound heterozygosity in KCNQ3, each inherited by a healthy parent; in this case, Kv7.2/Kv7.3 channels incorporating either KCNQ3 variant showed a smaller alteration in PIP_2_-dependent current regulation when compared to channels incorporating both KCNQ3 variants (Ambrosino et al., 2018). The fact that, in this latter case, parents were asymptomatic could be explained by the fact that, when compared to KCNQ2, KCNQ3 shows a greater “mutation tolerance,” as also suggested by the higher incidence of KCNQ2-DEE when compared to KCNQ3-DEE cases.

Altogether, the present results suggest that differences in both the alternative splicing process and in PIP_2_/CaM-dependent Kv7.2 current regulation may contribute to the divergent clinical phenotypes caused by the two KCNQ2 variants herein studied.

## Data Availability

The original contributions presented in the study are included in the article/Supplementary Material, further inquiries can be directed to the corresponding authors.
