# Supplementary figures and images for: Functional Characterization of Two Variants at the Intron 6—Exon 7 Boundary of the KCNQ2 Potassium Channel Gene Causing Distinct Epileptic Phenotypes
Source: Front Pharmacol. 2022 Jun 13;13:872645. doi: 10.3389/fphar.2022.872645 (PMC9234691; doi:10.3389/fphar.2022.872645)

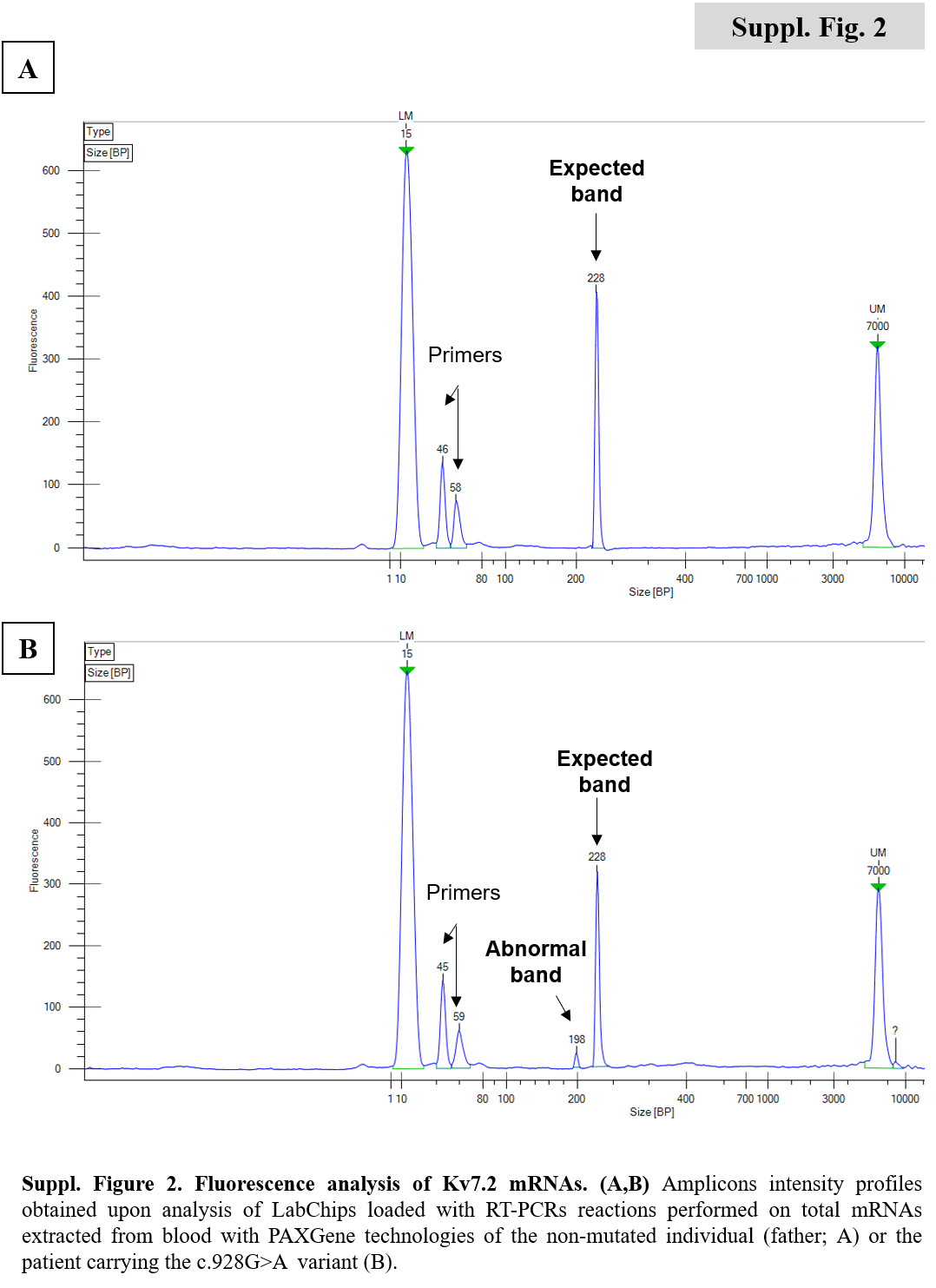

Supplement: Supplementary file 1 [file Image2.TIF]

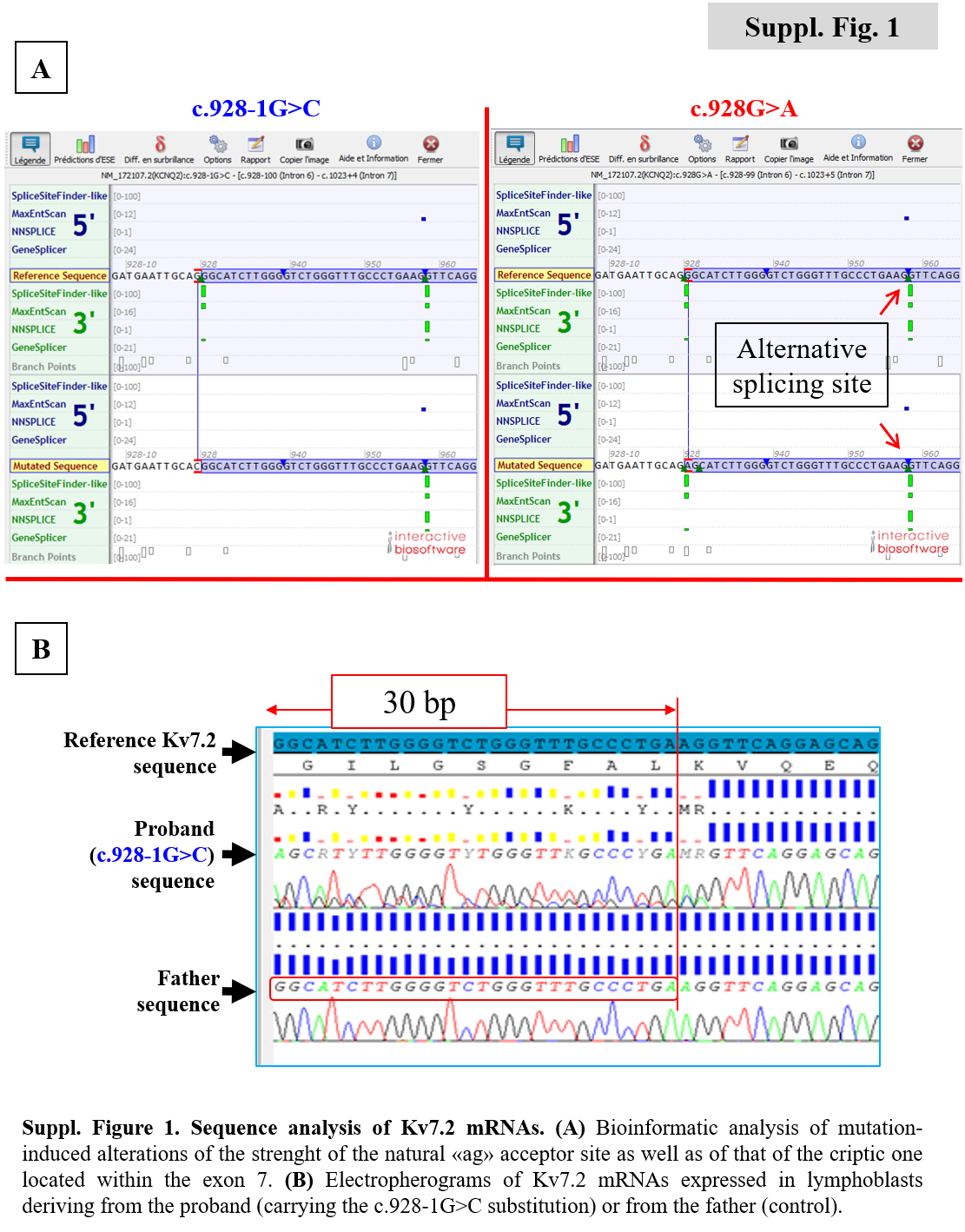

Supplement: Supplementary file 2 [file Image1.tif]
